# Comparative Genomics of Ecologically Differentiated *Nitrososphaera*-Related Ammonia-Oxidizing Archaea

**DOI:** 10.3390/microorganisms14102250

**Published:** 2026-10-04

**Authors:** Yuxiang Song, Zhongjun Jia

**Affiliations:** 1 State Key Laboratory of Soil and Sustainable Agriculture, Institute of Soil Science, Chinese Academy of Sciences, Nanjing 211135, China; 2 University of Chinese Academy of Sciences, Beijing 100049, China; 3 State Key Laboratory of Black Soils Conservation and Utilization, Northeast Institute of Geography and Agroecology, Chinese Academy of Sciences, Changchun 130102, China

**Keywords:** ammonia-oxidizing archaea, *Nitrososphaera*, NS-α, NS-δ, soil acidification, comparative genomics, DNA repair

## Abstract

Soil acidification strongly influences the ecology of terrestrial ammonia-oxidizing archaea (AOA). Within this group, the *Nitrososphaera*-related alpha (NS-α) and delta (NS-δ) lineages exhibit pronounced pH-associated ecological differentiation. In the preceding microcosm experiment, NS-α populations showed greater persistence than NS-δ during ammonium-driven soil acidification, yet the genomic basis underlying this ecological differentiation remains poorly understood. In this study, metagenomic sequencing of one near-neutral baseline microcosm (pH 7.35) and one acidified microcosm (pH 4.73) was used to reconstruct 12 *Nitrososphaera*-affiliated metagenome-assembled genomes (MAGs), including 2 NS-α and 10 NS-δ MAGs. These genomes were compared using genome-based phylogenetic analysis, *amoA* phylogeny, average amino acid identity (AAI), average nucleotide identity (ANI), and KEGG functional annotation to investigate lineage-level genomic differentiation and candidate functional genomic features. Genome-based phylogenetic and whole-genome similarity analyses supported clear differentiation between NS-α and NS-δ, while also revealing substantial genomic heterogeneity within NS-δ. Functions related to nitrogen acquisition and regulation, respiratory energy metabolism, autotrophic carbon fixation, and oxidative-stress defense were broadly distributed across both lineages and showed no clear NS-α-specific pattern. In contrast, Mre11, HerA, NurA, and RadA were detected in both reconstructed NS-α MAGs. Comparison with representative NS-α reference genomes, including the neutrophilic *Nitrososphaera viennensis* EN76, further showed that this DNA-repair-related gene repertoire is not restricted to acidic environments and is more consistent with a broader NS-α-associated genomic feature. Na^+^/H^+^ antiporters and Trk/Ktr-type K^+^ transport systems were not detected within either reconstructed NS-α MAG, although MAG-level non-detection was not interpreted as definitive lineage-level absence. Overall, the results identify genomic differences between NS-α and NS-δ but do not establish a functional advantage under acidification. The potential physiological significance of the DNA-repair-related pattern therefore remains to be experimentally tested.

## 1. Introduction

Soil acidification is increasingly recognized across terrestrial ecosystems and represents an important constraint on soil fertility, crop productivity, and biogeochemical functioning [1,2]. In agricultural soils, long-term application of ammonium-based fertilizers can accelerate acidification because nitrification releases protons and promotes nitrate-associated base-cation loss [3,4]. The first and often rate-limiting step of nitrification, ammonia oxidation, is carried out by ammonia-oxidizing bacteria (AOB), ammonia-oxidizing archaea (AOA), and complete ammonia oxidizers (comammox) [5,6]. AOA are widely distributed in soils and frequently make substantial contributions to ammonia oxidation under acidic conditions [7,8,9]. However, terrestrial AOA are phylogenetically and ecologically heterogeneous. Thaumarchaeota comprise a major archaeal lineage that includes many ammonia-oxidizing archaea and plays an important role in nitrification and nitrogen cycling in terrestrial ecosystems. Soil pH is one of the major factors shaping their global distribution, and the diversification of terrestrial Thaumarchaeota has been closely associated with adaptation to contrasting pH regimes [10].

Global *amoA*-based analyses further revealed pronounced ecological differentiation within major terrestrial AOA lineages [6]. Within the *Nitrososphaera*-related (NS) lineage, several *Nitrososphaera*-related alpha lineage (NS-α) subgroups are preferentially associated with acidic soils, whereas *Nitrososphaera*-related delta lineage (NS-δ) populations are more frequently detected in circumneutral soils [6]. This distribution suggests that substantial pH niche differentiation may occur even among relatively closely related NS populations [6]. Importantly, such ecological differentiation cannot necessarily be predicted from broad phylogenetic affiliation alone. Several characterized terrestrial AOA, including *Nitrososphaera viennensis* EN76, *Candidatus* (*Ca*.) Nitrososphaera evergladensis SR1, and *Nitrososphaera gargensis* Ga9.2, are generally regarded as neutrophilic representatives [11]. Nevertheless, Stable isotope probing (SIP) experiments have identified active *Nitrososphaera*-related AOA closely affiliated with the EN76 lineage in acidic soil [12]. These observations suggest that adaptation to low pH may emerge over relatively short evolutionary distances through fine-scale physiological or genomic differentiation.

Comparative genomic and physiological studies have identified several mechanisms that may contribute to acid adaptation in AOA [13]. Decreasing pH shifts the NH_3_/NH_4_^+^ equilibrium toward NH_4_^+^, thereby reducing the concentration of free NH_3_, which is generally considered the substrate directly available to ammonia monooxygenase [14,15]. Low external pH also increases the energetic cost of maintaining intracellular pH and ion homeostasis and may enhance oxidative and macromolecular stress [16,17]. High-affinity ammonium acquisition has therefore been proposed as an important feature supporting AOA activity under low-substrate conditions [13,18,19]. In the obligately acidophilic AOA *Nitrosotalea devanaterra* Nd1, genomic and transcriptomic analyses identified candidate adaptations involving ammonium transport, ion homeostasis, carbonic anhydrase, and membrane-associated traits [13]. In addition, horizontally acquired V-type-like ATPase operons have been associated with expansion of some terrestrial AOA into acidic habitats, suggesting a possible role for ATPase-mediated proton homeostasis [20].

However, most mechanistic insights into AOA acid adaptation have been derived from *Nitrosotalea* (NT), *Nitrosopumilus* (NP), or a limited number of cultivated terrestrial strains [13,21,22]. Much less is known about genomic differentiation within the environmentally abundant NS lineage itself. In particular, the contrasting environmental distributions of low-pH-associated NS-α populations and predominantly circumneutral NS-δ populations raise an important question: which genomic traits may contribute to their different ecological responses during soil acidification? Candidate mechanisms may involve not only substrate acquisition and proton homeostasis but also energy conservation, oxidative-stress defense, DNA damage repair, membrane properties, and broader ion regulation.

In the preceding soil microcosm experiment from which the samples analyzed here were selected, repeated NH_4_Cl addition was accompanied by a decline in soil pH from 7.35 to 4.73. NS-α and NS-δ showed contrasting ecological responses during this process, with the increase in NS-α accounting for most of the observed increase in the AOA community. DNA-SIP analysis further showed that the active AOA detected under NH_4_Cl treatment were predominantly affiliated with NS-α. These observations provided the ecological context for the present genome-resolved comparison. We therefore reconstructed *Nitrososphaera*-affiliated metagenome-assembled genomes (MAGs) from the near-neutral baseline and acidified microcosm samples and integrated phylogenomics, *amoA*-based phylogeny, average amino acid identity (AAI), average nucleotide identity (ANI), and comparative Kyoto Encyclopedia of Genes and Genomes (KEGG) annotation to characterize genomic differentiation between NS-α and NS-δ. Particular attention was given to functions related to nitrogen acquisition and regulation, respiratory energy metabolism, autotrophic carbon fixation, oxidative-stress defense, DNA damage repair, archaeal membrane metabolism, and ion homeostasis. We tested three hypotheses: (H1) NS-α and NS-δ are genomically differentiated; (H2) their contrasting ecological responses cannot be explained by a single functional category related to nitrogen acquisition, ATPase, or ion transport; and (H3) DNA-repair-related genes show a preferential genomic pattern in the reconstructed NS-α MAGs, while their physiological relevance during acidification remains to be functionally validated.

## 2. Materials and Methods

### 2.1. Soil Microcosm Incubation and Selection of Samples for Metagenomic Sequencing

Soil samples were collected from a long-term maize field at the Lishu Conservation Tillage Research and Development Station of the Institute of Applied Ecology, Chinese Academy of Sciences, Jilin Province, Northeast China (43°19′ N, 124°14′ E), from the 0–20 cm soil layer. The soil was classified as a black soil (Mollisol). A portion of the soil was air-dried and sieved for physicochemical characterization. Soil pH was measured in a soil–water suspension at a 1:2.5 (*w*/*w*) ratio using an FE28-Standard pH meter (Mettler Toledo Instrument, Shanghai, China). The initial soil pH was 7.35. Soil organic matter (SOM) and soil organic carbon (SOC) were determined using an HH-SA analyzer (Ronghua Instrument Manufacturing, Changzhou, China). Total nitrogen (TN) and hydrolysable nitrogen (HN) were measured using an ATN-300 instrument (CANY, Shanghai, China) and an LRH-250 instrument (YIHENG, Shanghai, China), respectively. Total phosphorus (TP) and available phosphorus (AP) were determined using a FIexA-200 spectrophotometer (ALPHA, Suzhou, China) and a UV 8000 spectrophotometer (METASH, Shanghai, China), respectively. Total potassium (TK) and available potassium (AK) were measured using an FP6431 analyzer (INESA, Shanghai, China).

Fresh soil equivalent to 5.0 g dry weight was transferred to 120 mL serum bottles, adjusted to 60% of the maximum water-holding capacity, and pre-incubated at 28 °C in the dark for 10 days. The NH_4_Cl treatment subsequently received 100 μg NH_4_Cl-N g^−1^ dry soil per week. To maintain oxic conditions, the bottles were flushed weekly with pressurized synthetic air (20% O_2_ and 80% N_2_).

For the metagenomic analysis in the present study, two individual microcosms were selected. One was sampled at the end of the 10-day pre-incubation period, before NH_4_Cl addition, and represented the near-neutral baseline condition (pH 7.35). The second was sampled after 60 days of repeated NH_4_Cl addition, when soil pH had decreased to 4.73, and represented the acidified condition. One individual microcosm per condition (*n* = 1) was used for metagenomic sequencing. The two samples were processed and sequenced separately and were not pooled.

Total genomic DNA was extracted from each of the two selected microcosm samples using the FastDNA SPIN Kit (MP Biomedicals, Cleveland, OH, USA) according to the manufacturer’s instructions. One DNA extraction was performed for each selected microcosm sample, and the DNA extracts were not pooled prior to library preparation. DNA concentration and purity were assessed using a NanoDrop 2000 spectrophotometer (Thermo Scientific, Wilmington, DE, USA), and DNA integrity was examined by electrophoresis on a 1% agarose gel. Metagenomic libraries were prepared using the TruSeq^TM^ DNA Sample Preparation Kit (Illumina, San Diego, CA, USA) and sequenced on an Illumina HiSeq platform by Majorbio Bio-Pharm Technology Co., Ltd. (Shanghai, China). Each sample yielded more than 31.1 Gb of raw sequence data, with a total of 62.3 Gb obtained across the two samples. The Q30 values of the raw reads exceeded 95.5%.

Metagenomic sequencing generated 150 bp paired-end reads. No additional read trimming or quality filtering was performed before assembly. The two metagenomic samples were processed independently and assembled separately rather than co-assembled. Paired-end reads from each sample were assembled de novo using MEGAHIT v1.2.9 [23] with the parameters --kmin-1pass and --mem-flag 2. The resulting contigs were binned independently using MaxBin v2.2.7 [24], with a minimum contig length of 1000 bp. Protein-coding genes were predicted using Prodigal v2.6.3 in metagenomic mode (-p meta) with complete-gene prediction (-c) and genetic code 11 (-g 11). The initially reconstructed bins were further refined based on contig-level taxonomic assignments. Contigs assigned to bacterial or eukaryotic taxa were removed from the corresponding archaeal bins. The refined MAGs were subsequently reassessed using CheckM2 v1.1.0 [25] to estimate genome completeness and contamination. Although refinement reduced estimated contamination in several MAGs, residual contamination remained >10% in five MAGs (bin043, bin017, bin022, bin024, and bin079). The refined MAG assemblies were used for the revised functional annotation and comparative analyses. Taxonomic classification of the recovered MAGs was performed using GTDB-Tk v2.4.0 [26] against GTDB release R226 with the --skip_ani_screen option.

MAGs assigned to the *Nitrososphaera*-related lineage were retained for subsequent phylogenetic and comparative genomic analyses. A total of 12 *Nitrososphaera*-affiliated MAGs were reconstructed from the two metagenomic samples described above. Eight MAGs (bin002, bin003, bin008, bin013, bin017, bin022, bin024, and bin043) were recovered from the near-neutral baseline microcosm collected after the 10-day pre-incubation and before NH_4_Cl addition (pH 7.35). The remaining four MAGs (bin072, bin079, bin088, and bin112) were recovered from the acidified microcosm collected after 60 days of repeated NH_4_Cl addition (pH 4.73). Based on subsequent phylogenomic analyses, bin043 and bin112 were assigned to NS-α, whereas the remaining ten MAGs were assigned to NS-δ.

### 2.2. Genome-Based Phylogenetic Analysis

Representative AOA genomes covering the major phylogenetic lineages relevant to the reconstructed MAGs were retrieved from the NCBI database and included as reference genomes; accession identifiers are listed in Supplementary Table S1. Genome-based phylogenetic relationships were inferred using the GTDB-Tk archaeal marker set (ar53), comprising 53 archaeal-specific single-copy protein markers from GTDB release R226. Marker proteins were identified and aligned using the standard GTDB-Tk v2.4.0 workflow before concatenation. The concatenated alignment was trimmed using the canonical GTDB-Tk archaeal mask, resulting in a final alignment of 8062 amino-acid positions. Maximum-likelihood phylogenetic reconstruction was performed using IQ-TREE v1.6.12 [27]. The optimal amino acid substitution model was selected automatically using ModelFinder implemented in IQ-TREE. Statistical support for individual branches was evaluated using 1000 ultrafast bootstrap replicates and 1000 SH-like approximate likelihood ratio test replicates with the parameters -m MFP -bb 1000 -alrt 1000 -nt AUTO. The reconstructed MAGs were assigned to NS-α, NS-δ, and other major AOA lineages according to their phylogenetic positions relative to representative reference genomes. *Methanobrevibacter smithii* ATCC 35061 (GCA_000016525) was selected a priori as a taxonomically external archaeal lineage, and the final tree was rooted on the branch leading to this genome. The final phylogenetic tree was visualized and annotated using the Interactive Tree of Life (iTOL) (v7) [28].

### 2.3. amoA Phylogenetic Analysis

To provide an independent phylogenetic assessment based on the functional marker gene for ammonia oxidation, confidently identified *amoA* sequences recovered from the refined MAGs were analyzed together with representative AOA *amoA* sequences and previously reported NS-α and NS-δ sequences (Supplementary Table S1). Because the reconstructed MAGs varied in completeness, failure to recover an *amoA* sequence from a MAG was not interpreted as evidence of biological absence. Candidate *amoA* assignments were reassessed after MAG refinement using contig retention and revised high-confidence KOfamScan profile-HMM annotations, and only sequences retained in the final revised analysis were used for phylogenetic reconstruction (Supplementary Table S2).

Multiple sequence alignment was performed using the MAFFT web service provided by EMBL-EBI Job Dispatcher (https://www.ebi.ac.uk/jdispatcher/msa/mafft, accessed on 30 September 2026), and the resulting alignment was used to construct a maximum-likelihood phylogenetic tree using IQ-TREE v1.6.12 [27]. The best-fitting nucleotide substitution model was selected by BIC (SYM+I+G4), and branch support was estimated from 1000 bootstrap replicates. The resulting tree was visualized and annotated using iTOL (v7) [28].

### 2.4. ATPase Subunit D Phylogenetic Analysis

To investigate the phylogenetic diversity of ATPase subunit D homologs, ATPase subunit D nucleotide sequences retained after MAG refinement were analyzed together with homologous sequences from representative AOA genomes (Supplementary Table S1). Multiple sequence alignment was performed using the same MAFFT web service, and the resulting alignment was used to construct a maximum-likelihood phylogenetic tree using IQ-TREE v1.6.12 [27] under the BIC-selected TIM3+F+G4 model with 1000 bootstrap replicates. The phylogenetic positions of the MAG-derived ATPase subunit D sequences were compared with representative reference sequences to distinguish A-type-like and V-type-like groups. The resulting tree was visualized and annotated using iTOL [28].

### 2.5. Average Nucleotide Identity and Average Amino Acid Identity Analyses

Genome-level relatedness among the reconstructed *Nitrososphaera* MAGs and representative AOA genomes was further evaluated using ANI and AAI. Pairwise ANI values were calculated using FastANI v1.33 [29]. Genome assemblies were provided in FASTA format and subjected to completeness and contamination assessment before ANI analysis. All-versus-all comparisons were performed using genome query and reference lists specified with the --ql and --rl options. The resulting pairwise ANI estimates were compiled into a symmetric similarity matrix. Because FastANI is primarily intended for genome comparisons within approximately the 70–100% nucleotide identity range, estimates from highly divergent genome pairs were interpreted cautiously. Comparisons for which FastANI did not return a reliable estimate were retained as missing values rather than being interpreted as zero similarity. For each comparison, the alignment fraction was considered together with the ANI value when evaluating genome-wide relatedness.

AAI was calculated independently using a reciprocal best-hit approach based on DIAMOND v2.1.4 [30]. Protein sequences encoded by each genome were extracted to generate genome-specific proteomes, and an individual DIAMOND database was constructed for each proteome. For each pair of genomes, bidirectional DIAMOND BLASTP searches were conducted using an *E*-value threshold of 1 × 10^−5^, with only the highest-scoring match retained for each query protein.

Reciprocal best hits (RBHs) were identified by matching the top-hit relationships obtained from the two search directions. For each genome pair, AAI was calculated as the arithmetic mean of the percentage amino acid identities of all identified RBH protein pairs. Pairwise AAI values were subsequently organized into a symmetric matrix for comparison and visualization. ANI and AAI were used as complementary genome-wide measures to assess genomic relatedness among NS-α, NS-δ, and representative AOA genomes rather than as independent criteria for lineage assignment.

### 2.6. Functional Genome Annotation and Comparative Analysis

Protein-coding genes predicted from the refined MAGs were functionally annotated using KOfamScan v1.3.0 [31] against the KEGG Orthology profile database, with an *E*-value threshold of 1 × 10^−5^. For the revised comparative analysis, only KO assignments with *E*-values ≤ 1 × 10^−5^, scores exceeding the corresponding KO-specific thresholds, and high-confidence assignments marked with an asterisk (*) in the KOfamScan output were retained. To further validate the DNA-repair-related annotations, the predicted Mre11, HerA, and NurA proteins from bin043 and bin112 were independently analyzed using the NCBI Conserved Domain Search (CDD), with an *E*-value cutoff of 0.01 and composition-corrected scoring. Gene coordinates and strand orientations were obtained from the Prodigal GFF annotations, and the genomic organization of the corresponding loci was examined (Figure S1; Tables S7 and S8). Available contig-level taxonomic assignments and contig/MAG GC characteristics were considered when evaluating genomic consistency; contig-specific read depth/coverage was not retained and is therefore reported as unavailable rather than replaced by MAG-level coverage (Tables S7 and S8). In addition, the apparent non-detection of Rad50 in the NS-α MAGs was reassessed by examining the complete non-redundant protein catalogs generated from all assembled contigs of the corresponding source metagenomes (Table S3). Homologs detected only at the whole-metagenome level were not assigned to individual MAGs without independent evidence of genomic affiliation. The distribution of 29 selected functional categories was then re-evaluated across the refined NS-α and NS-δ MAGs. Because residual contamination remained >10% in several MAGs after refinement, an additional sensitivity analysis was performed by excluding MAGs with estimated contamination >10%. This filtering retained seven MAGs, comprising bin112 from NS-α and bin002, bin003, bin008, bin013, bin072, and bin088 from NS-δ. Because only one NS-α MAG remained after this stringent filtering, the contamination-filtered subset was used to assess the sensitivity of individual functional patterns to residual contamination rather than as the sole basis for lineage-level comparison.

Based on their potential relevance to ammonia oxidation and cellular responses to soil acidification, 29 enzyme or functional protein categories were selected for focused comparison. These categories encompassed ammonia oxidation and nitrogen acquisition, nitrogen regulation and urease-mediated nitrogen utilization, respiratory energy metabolism, V/A-type ATPase, the archaeal 3-hydroxypropionate/4-hydroxybutyrate (3-HP/4-HB) carbon fixation pathway, oxidative-stress defense, DNA damage repair and homologous recombination, archaeal membrane-lipid metabolism, and ion homeostasis. The MAG × 29-category matrix and KO identifiers are provided in Supplementary Table S4, and the underlying gene- and subunit-level high-confidence annotations are provided in Supplementary Table S5.

For each MAG, the number of high-confidence genes assigned to each selected functional category was recorded. For multi-subunit systems, including ammonia monooxygenase, urease, respiratory complexes, and ATPase, the reported values represent the summed number of annotated subunit genes rather than the number of complete enzyme complexes or pathways. Functional comparisons therefore emphasized high-confidence detections and consistent co-occurrence patterns across MAGs rather than treating non-detection as definitive biological absence.

Because the reconstructed MAGs differed substantially in completeness and estimated contamination, absolute gene counts were interpreted cautiously and were not used alone to infer lineage-specific functional enrichment. Instead, greater emphasis was placed on functional patterns consistently observed in both NS-α MAGs and their distribution among the ten NS-δ MAGs. These comparisons were used to identify candidate genomic features potentially associated with the contrasting ecological responses of NS-α and NS-δ during ammonium-driven soil acidification. The resulting functional patterns were interpreted as genomic potential rather than direct evidence of gene expression or physiological activity.

To assess whether the DNA-repair-related pattern observed in the reconstructed NS-α MAGs represented a broader lineage-associated feature, three representative NS-α reference genomes—*Nitrososphaera viennensis* EN76, *Ca*. Nitrososphaera gargensis Ga9.2, and *Ca*. Nitrososphaera evergladensis SR1—were additionally examined (Supplementary Table S6). Homologs of Mre11, HerA, NurA, and RadA, together with candidate Rad50/SbcC-like homologs, were screened, and the genomic organization of regions containing Mre11, HerA, and NurA was compared with the corresponding regions in bin043 and bin112.

## 3. Results

### 3.1. Phylogenomic Classification of Reconstructed NS MAGs

**Figure 1:**
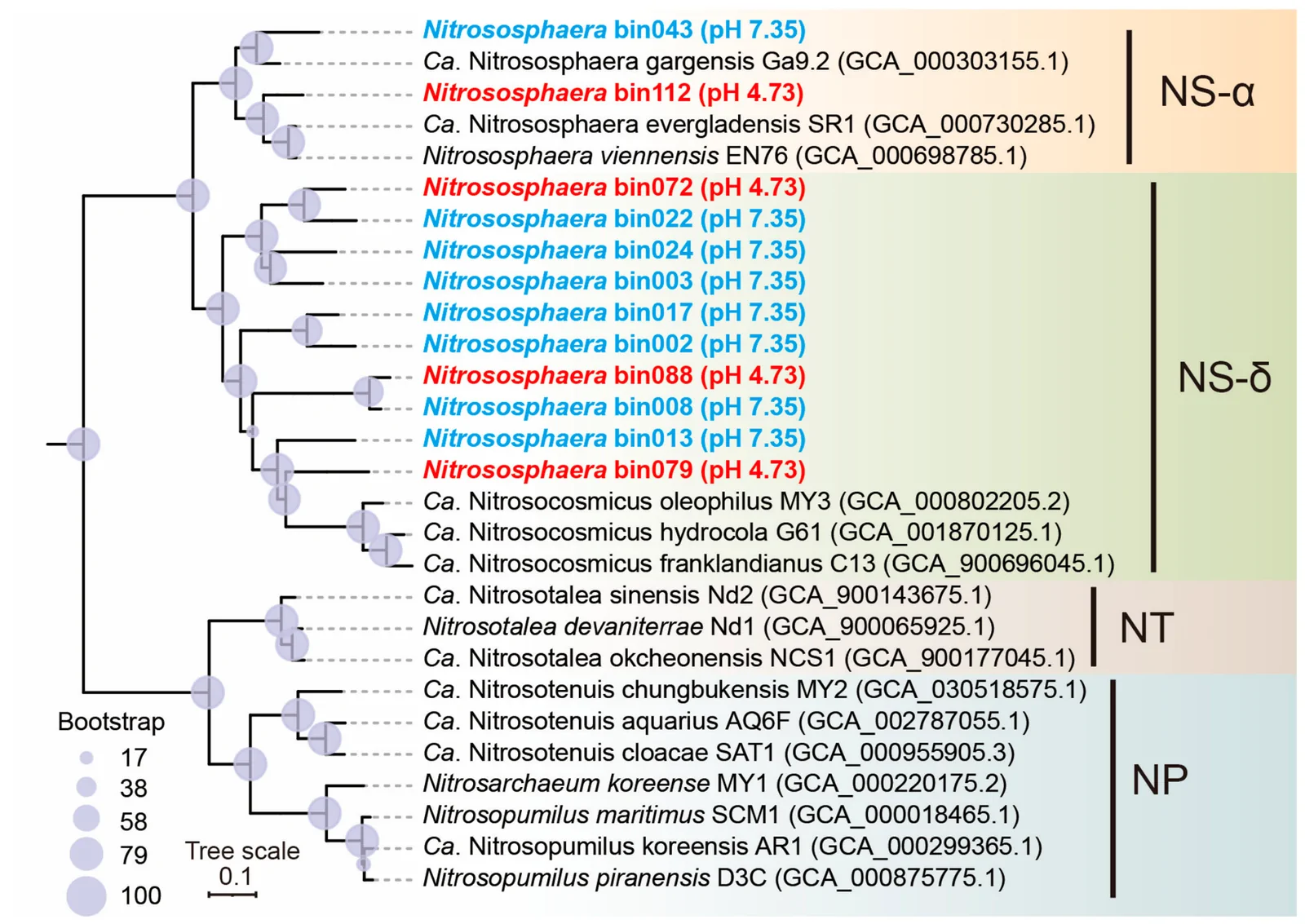
Phylogenomic placement of reconstructed *Nitrososphaera*-affiliated metagenome-assembled genomes (MAGs). The maximum-likelihood phylogenomic tree was reconstructed from the GTDB-Tk ar53 marker set using IQ-TREE v1.6.12, with the best-fitting amino acid substitution model selected by ModelFinder. Branch support was evaluated using 1000 ultrafast bootstrap and 1000 SH-aLRT replicates; circle size represents bootstrap support, and values >50% were treated as supported. MAGs recovered from the near-neutral baseline microcosm (pH 7.35) are shown in blue, whereas those recovered from the acidified microcosm (pH 4.73) are shown in red. Reference genome accessions are listed in Supplementary Table S1.

A total of 12 *Nitrososphaera*-affiliated MAGs were reconstructed from the near-neutral baseline and acidified microcosm samples and retained for comparative genomic analysis. Genome-based phylogenetic reconstruction resolved these MAGs into two major NS lineages (Figure 1). Bin043, reconstructed from the near-neutral baseline microcosm (pH 7.35), and bin112, recovered from the acidified microcosm (pH 4.73), clustered within NS-α together with representative *Nitrososphaera* genomes, including *Ca*. Nitrososphaera gargensis Ga9.2, *Ca*. Nitrososphaera evergladensis SR1, and *Nitrososphaera viennensis* EN76. In contrast, bin002, bin003, bin008, bin013, bin017, bin022, bin024, bin072, bin079, and bin088 were assigned to NS-δ. Among the four MAGs recovered from the acidified microcosm, bin112 belonged to NS-α, whereas bin072, bin079, and bin088 belonged to NS-δ. The phylogenetic separation of the reconstructed genomes provided the basis for subsequent comparisons between NS-α and NS-δ.

**Figure 2:**
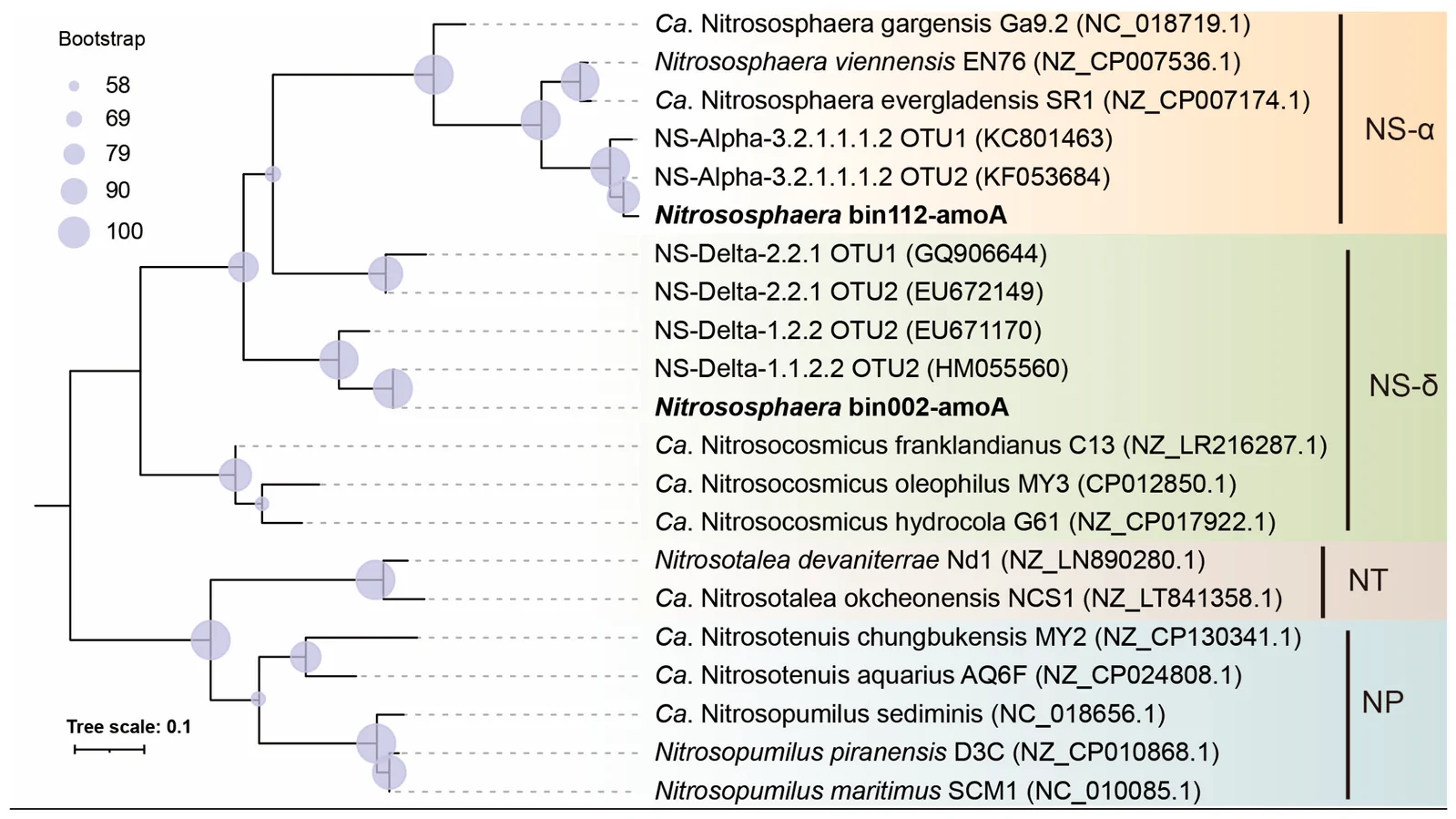
Phylogenetic placement of *amoA* sequences retained after MAG refinement and reassessment. The maximum-likelihood tree was constructed using retained MAG-derived *amoA* sequences together with representative AOA *amoA* sequences and previously reported NS-α and NS-δ OTUs. MAG-derived *amoA* sequences are shown in bold. The tree was constructed using IQ-TREE v1.6.12 with the BIC-selected SYM+I+G4 model, and branch support was estimated from 1000 bootstrap replicates; bootstrap values ≥ 50% are shown. Reference sequence accessions are provided in Supplementary Table S1.

The genome-based classification was further evaluated using *amoA*, the functional marker gene for ammonia oxidation. In the current revised analysis, confidently retained MAG-derived *amoA* sequences from bin112 and bin002 were included in the final tree. The bin112 *amoA* sequence clustered within NS-α together with previously described NS-α and representative *Nitrososphaera* sequences, whereas the bin002 *amoA* sequence grouped with NS-δ sequences (Figure 2; Supplementary Table S2). The two sequences originally treated as *amoA* candidates in bin022 were excluded after refinement/reannotation. Because MAG completeness varied, non-recovery of *amoA* from other MAGs was not interpreted as definitive biological absence.

### 3.2. Genome Characteristics of Reconstructed Nitrososphaera MAGs

Following contig-level refinement and re-evaluation with CheckM2 v1.1.0, the 12 reconstructed MAGs continued to vary in genome quality and genome characteristics (Table 1). Estimated completeness ranged from 29.5% to 79.5%, reconstructed genome sizes ranged from 0.84 to 4.77 Mb, and estimated contamination ranged from 2.8% to 24.8%. The two NS-α MAGs, bin043 and bin112, had reconstructed genome sizes of 1.37 and 1.52 Mb and estimated contamination values of 15.4% and 7.42%, respectively. Greater variation in genome size remained evident among the NS-δ MAGs. Because genome completeness and residual contamination still varied among the MAGs, subsequent functional comparisons emphasized high-confidence detections and consistent co-occurrence patterns rather than treating non-detection as definitive absence or relying on absolute gene copy number. Findings involving MAGs with residual contamination > 10% were interpreted cautiously and were further evaluated in a contamination-filtered sensitivity analysis.

### 3.3. Genome-Wide Relatedness Among NS-α and NS-δ MAGs

**Figure 3:**
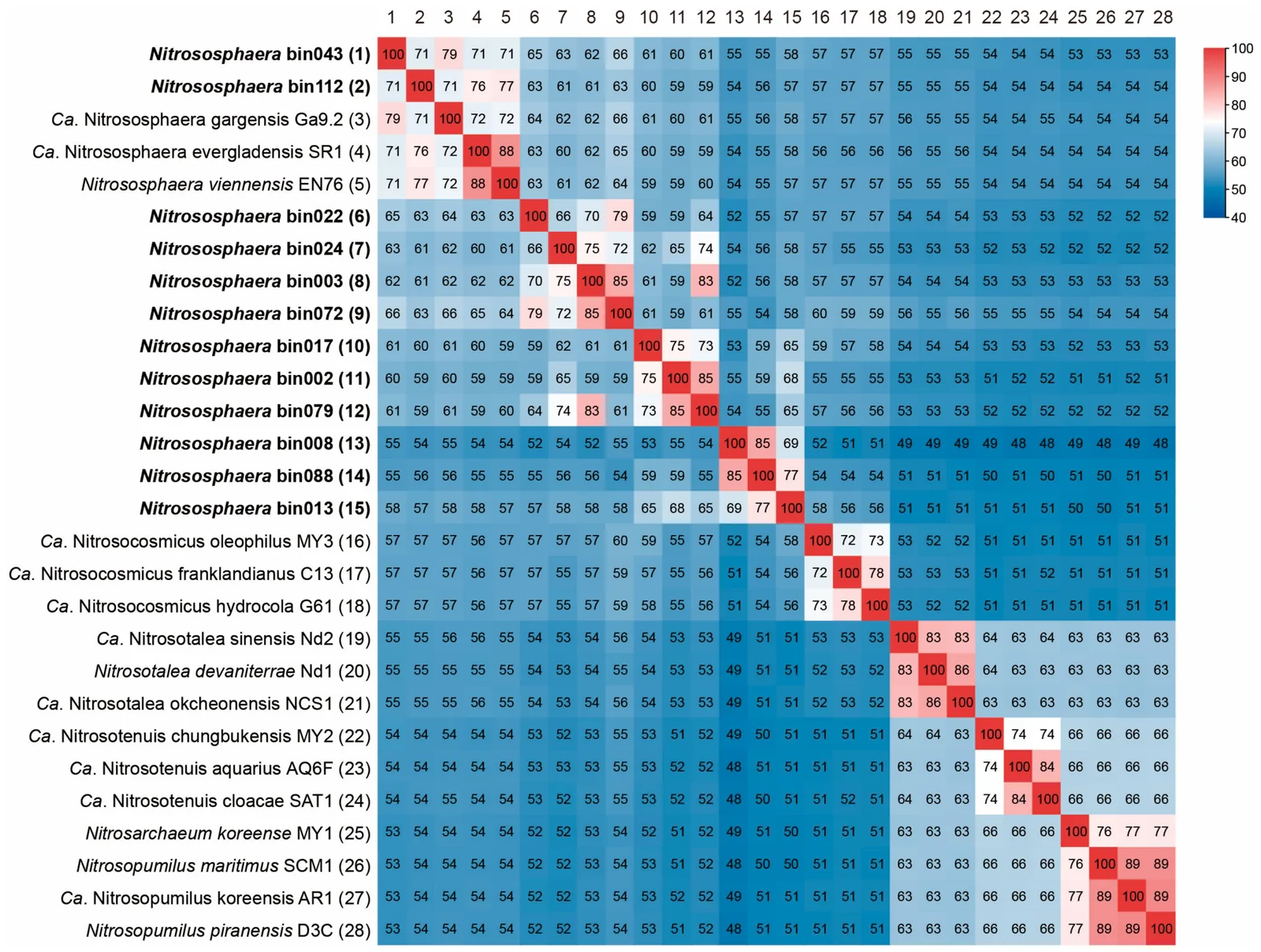
Pairwise average amino acid identity (AAI) among reconstructed AOA MAGs and representative reference genomes. The heatmap shows pairwise AAI values (%) among 12 reconstructed *Nitrososphaera*-affiliated MAGs and representative genomes from major AOA lineages. Names of reconstructed MAGs are shown in bold. The two NS-α MAGs are bin043 and bin112, whereas the remaining reconstructed MAGs belong to NS-δ. Numbers within cells indicate pairwise AAI values.

AAI analysis further supported the genomic differentiation between NS-α and NS-δ (Figure 3). The two NS-α MAGs, bin043 and bin112, shared approximately 70% AAI and showed higher similarity to representative NS-α genomes (approximately 70–78%) than to most NS-δ MAGs (approximately 54–65%). Considerable heterogeneity was observed within NS-δ, with some genome pairs showing high AAI, such as bin002–bin079 (93%) and bin008–bin088 (89%), whereas others were substantially less similar.

**Figure 4:**
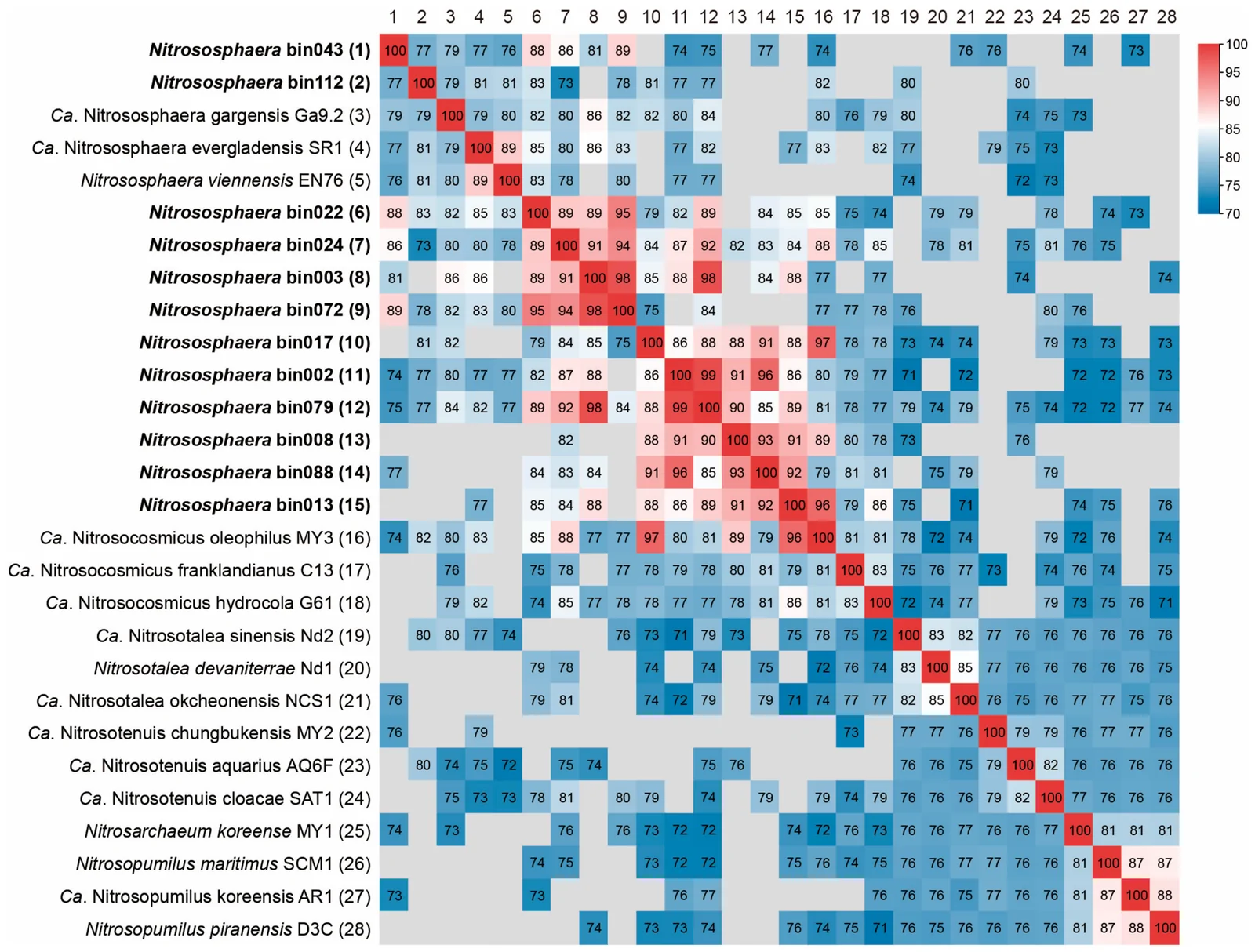
Pairwise average nucleotide identity (ANI) among reconstructed AOA MAGs and representative reference genomes. The heatmap shows pairwise ANI values (%) among the reconstructed *Nitrososphaera*-affiliated MAGs and representative genomes from major AOA lineages. Names of reconstructed MAGs are shown in bold. Gray cells indicate genome pairs for which no reliable ANI estimate was retained and were treated as missing values.

ANI analysis provided complementary nucleotide-level resolution of genome relatedness (Figure 4). FastANI returned an approximate ANI value of 77% for the two NS-α MAGs, indicating substantial nucleotide-level divergence between bin043 and bin112. This value was interpreted together with the corresponding alignment fraction rather than being used alone to infer overall genome-wide relatedness. In contrast, several NS-δ genome pairs showed high ANI values of approximately 98–99%, consistent with closely related genomic populations within NS-δ. For more divergent genome pairs, FastANI did not provide estimates considered sufficiently reliable for quantitative interpretation; these comparisons are shown as gray cells in Figure 4 and were treated as missing values rather than as low ANI values. Alignment fractions were considered together with ANI values when evaluating genome relatedness. Overall, the combined ANI, alignment-fraction, and AAI results indicate substantial genomic heterogeneity among the reconstructed *Nitrososphaera* MAGs, particularly within NS-δ.

### 3.4. Comparative Functional Genomic Patterns in NS-α and NS-δ

Comparative functional analysis of the refined MAGs was performed using high-confidence KOfamScan annotations to examine 29 functional categories potentially relevant to ammonia oxidation and cellular responses to soil acidification (Supplementary Table S4). These categories encompassed nitrogen acquisition and regulation, respiratory energy metabolism, the 3-hydroxypropionate/4-hydroxybutyrate (3-HP/4-HB) carbon fixation pathway, oxidative-stress defense, DNA repair, archaeal membrane-lipid metabolism, and ion homeostasis. Only KOfamScan assignments satisfying all three criteria—an *E*-value ≤ 1 × 10^−5^, a score exceeding the corresponding KO-specific threshold, and designation with an asterisk (*) in the KOfamScan output—were retained in the revised comparison; gene- and subunit-level assignments are listed in Supplementary Table S5. Functional comparisons therefore emphasized high-confidence detections and consistent co-occurrence patterns across the refined MAGs rather than differences in absolute annotation counts. Given variation in MAG completeness and residual contamination, non-detection of a gene or functional category was interpreted cautiously and was not considered definitive evidence of biological absence.

To provide a more detailed comparison of nitrogen-related genomic potential, genes associated with ammonia oxidation, ammonium acquisition, nitrogen regulation, and urea utilization were examined separately (Supplementary Tables S4 and S5). High-confidence AMO-related subunit annotations were detected in NS-α bin112 and in several NS-δ MAGs, whereas no high-confidence AMO-related annotation was recovered from bin043. Ammonium transporter (Amt) genes were retained in both NS-α MAGs and were also detected in several NS-δ MAGs. High-confidence P-II nitrogen-regulatory and urease-associated annotations were detected in bin112 but not in refined bin043 and were likewise present in several NS-δ MAGs. No high-confidence HAO-like annotation was detected in any of the refined MAGs under the applied annotation criteria. Overall, these nitrogen-related functions varied both between and within the two NS lineages rather than showing a consistent NS-α-specific distribution.

Similarly, genes associated with respiratory complexes, V/A-type ATPase, and enzymes of the 3-HP/4-HB carbon fixation pathway were distributed across both NS-α and NS-δ MAGs. Thus, no consistent NS-α-specific pattern was evident for nitrogen acquisition and regulation, respiratory energy conservation, or autotrophic carbon fixation.

**Figure 5:**
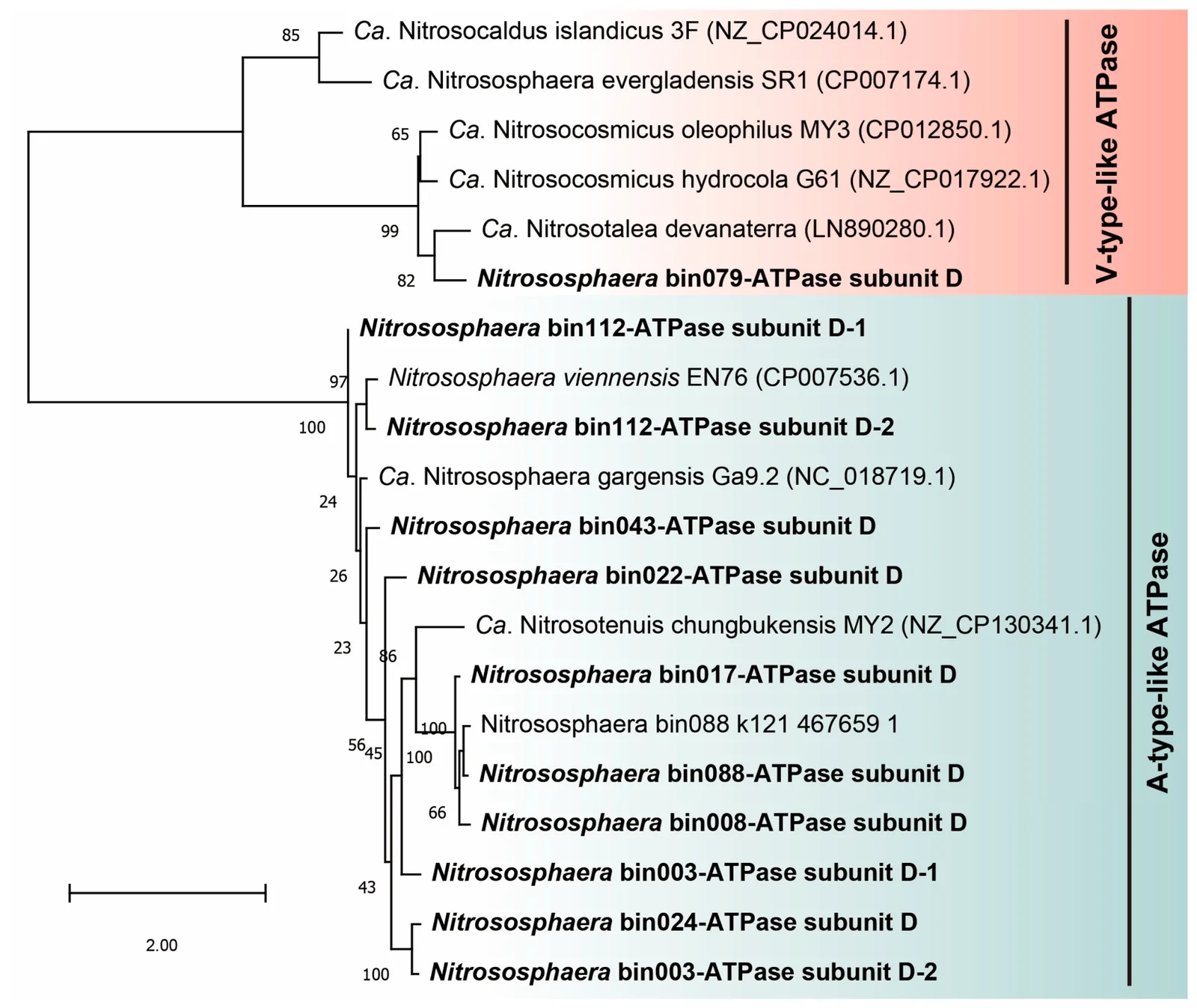
Phylogenetic relationships of ATPase subunit D homologs retained after MAG refinement and representative AOA homologs. MAG-derived sequences are shown in bold. The maximum-likelihood tree was constructed using IQ-TREE v1.6.12 under the BIC-selected TIM3+F+G4 model with 1000 bootstrap replicates. Bootstrap values are shown at internal nodes. Because the tree is based on subunit D alone, it does not establish the presence of complete A-type or V-type ATPase operons. Reference accessions are listed in Supplementary Table S1.

Phylogenetic analysis of ATPase subunit D resolved the retained sequences into A-type-like and V-type-like groups (Figure 5). Following contig-level refinement and high-confidence reannotation, only one ATPase subunit D homolog was retained in the NS-δ MAG bin079, and this homolog grouped within the V-type clade. All ATPase D homologs recovered from the two NS-α MAGs, bin043 and bin112, were positioned within the A-type group, together with homologs from representative *Nitrososphaera* genomes such as *Nitrososphaera viennensis* EN76 and *Ca*. Nitrososphaera gargensis Ga9.2 (Supplementary Table S1). Most ATPase D homologs from the remaining NS-δ MAGs also belonged to the A-type group. These results did not support a preferential association of V-type ATPase D homologs with NS-α. Because the analysis is based on subunit D and the refined bin079 ATPase-related subunits are distributed across separate contigs, a complete continuous ATPase operon could not be reconstructed for bin079 (Supplementary Table S7).

Multiple oxidative-stress-related functions were retained in the refined NS MAGs (Supplementary Tables S4 and S5). Catalase- and peroxiredoxin-associated genes were detected in both NS-α MAGs, whereas a high-confidence SOD annotation was retained only in bin112. Comparable antioxidant functions were also detected in several NS-δ MAGs. Thus, oxidative-stress defense remained broadly distributed and was not specific to NS-α. Genomic-context information for the catalase candidates is provided in Supplementary Table S7.

A notable DNA-repair-related genomic pattern involved Mre11, HerA, NurA, and RadA. These genes were retained in both refined NS-α MAGs. Mre11, HerA, and NurA occurred in compact adjacent regions with the same transcriptional gene order (NurA–HerA–Mre11) in bin043 and bin112, whereas RadA was located on a separate contig in each MAG (Figure S1; Supplementary Table S8). Independent conserved-domain analysis supported the Mre11, HerA, and NurA assignments, and contig-level GC/taxonomic context is summarized in Supplementary Table S7. In the revised MAG annotations, HerA and NurA were not detected in the NS-δ MAGs, whereas Rad50 was detected only in bin002 (Supplementary Tables S4 and S5). Broader screening of the complete metagenomic protein catalogs recovered additional HerA, NurA, and Rad50 homologs, but these could not be confidently assigned to individual NS MAGs (Supplementary Table S3); therefore, MAG-level non-detection was not interpreted as definitive gene absence. Comparison with three representative NS-α reference genomes showed that Mre11, HerA, NurA, and RadA homologs were also present in *Nitrososphaera viennensis* EN76, *Ca*. Nitrososphaera gargensis Ga9.2, and *Ca*. Nitrososphaera evergladensis SR1 (Supplementary Table S6). Their occurrence in the neutrophilic *Nitrososphaera viennensis* EN76 indicates that this gene repertoire is not restricted to acidic environments. We therefore interpret it as a broader NS-α-associated genomic pattern rather than an acid-specific innovation.

Membrane-lipid-associated functions showed variable distributions after refinement (Supplementary Tables S4 and S5). AIPS was retained in bin112 but was not detected as a high-confidence annotation in refined bin043 and was also present in several NS-δ MAGs. Other membrane-lipid-associated functions, including tetraether lipid synthase, glycerol-1-phosphate dehydrogenase, and geranylgeranyl/polyprenyl synthase, also showed variable distributions among the refined MAGs. No high-confidence Na^+^/H^+^ antiporter or Trk/Ktr-type K^+^ transport annotations were detected within either refined NS-α MAG, whereas one or both transporter categories were detected in several NS-δ MAGs (Supplementary Tables S4, S5 and S7). Additional screening of the complete metagenomic protein catalogs identified both Na^+^/H^+^ antiporter and Trk/Ktr-type transporter homologs in the pH 7.35 and pH 4.73 source metagenomes (Supplementary Table S3). These community-level hits could not be confidently assigned to the reconstructed NS-α MAGs or to specific NS lineages based on KO identity alone; thus, non-detection in bin043 and bin112 was interpreted as MAG-level non-detection rather than definitive lineage-level absence.

**Figure 6:**
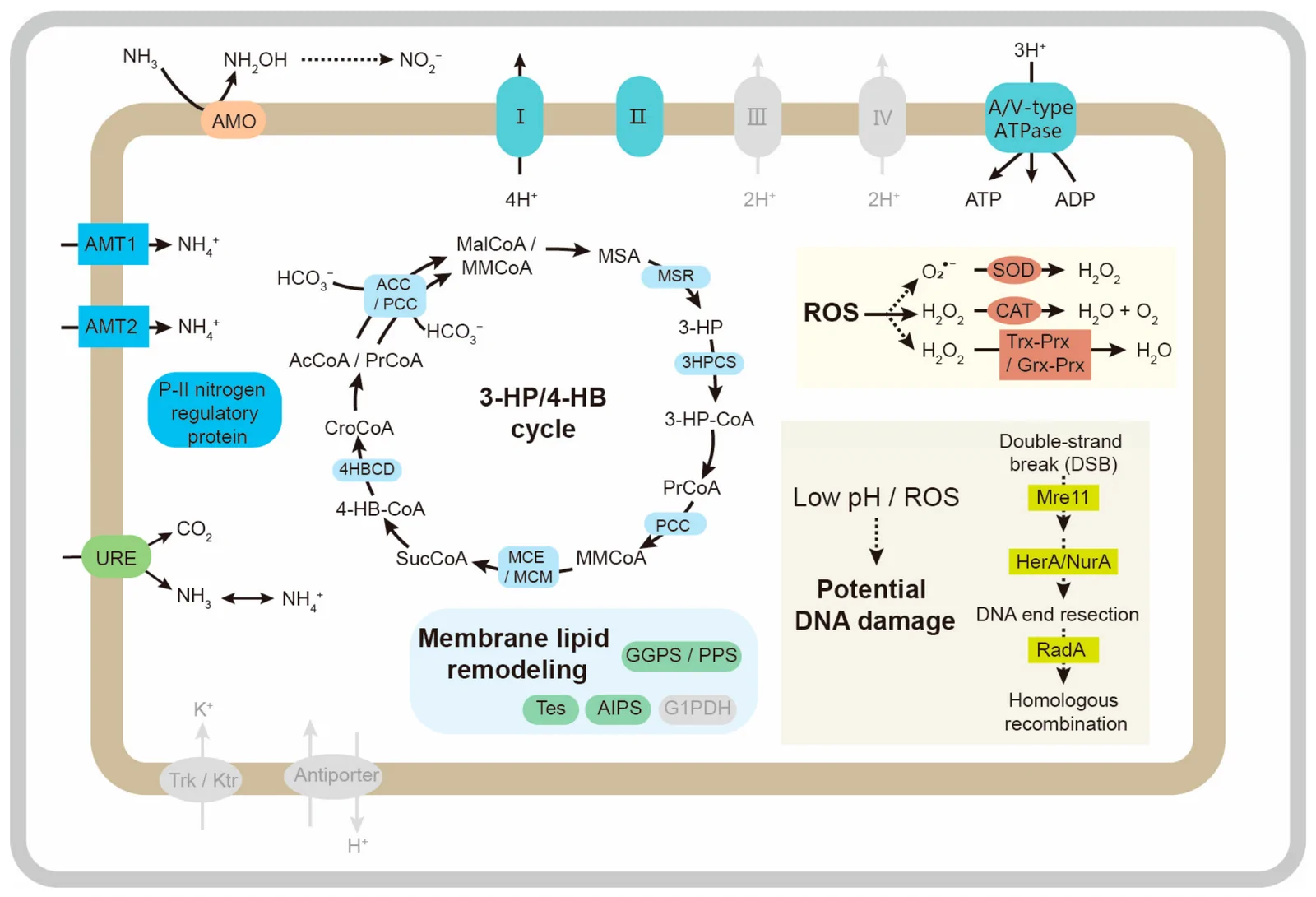
Schematic summary of genomic functions examined in relation to NS-α and NS-δ differentiation during ammonium-driven soil acidification. Solid colored symbols indicate functions supported by high-confidence annotations in at least one reconstructed NS-α MAG. Gray/crossed symbols indicate functions not detected in either reconstructed NS-α MAG under the applied high-confidence criteria. Dashed arrows and dashed outlines denote hypothesized physiological relationships inferred from established biological functions and are not experimentally validated in this study. The DNA-repair portion summarizes the detected Mre11–HerA–NurA–RadA repertoire; because Rad50 was not detected in either NS-α MAG, the diagram should not be interpreted as a complete classical Mre11–Rad50 double-strand-break repair pathway. The schematic summarizes genomic annotations and candidate functional relationships and should not be interpreted as a confirmed physiological pathway or acid-adaptation mechanism.

Overall, the functional comparison did not identify a single canonical acid-resistance pathway uniquely associated with NS-α. Most examined functions were shared or variably distributed across the two lineages. The Mre11–HerA–NurA–RadA repertoire represented a notable DNA-repair-related genomic pattern in the two reconstructed NS-α MAGs, but its physiological relevance during acidification remains hypothetical (Figure 6).

## 4. Discussion

### 4.1. Ecological Differentiation Between NS-α and NS-δ and Its Ecological Implications

Soil pH is a major determinant of terrestrial AOA distribution and has been closely linked to the evolutionary diversification of Thaumarchaeota [10]. Global *amoA*-based analyses indicate that several NS-α subgroups are preferentially associated with acidic soils, whereas NS-δ is more frequently detected in circumneutral environments [6]. These patterns suggest that substantial pH niche differentiation can occur within relatively closely related NS populations. Our previous incubation study provided the ecological context for investigating this differentiation, showing that ammonium chloride-induced soil acidification from pH 7.35 to pH 4.73 was accompanied by a community shift favoring NS-α over NS-δ. In the present genome-resolved analysis, bin043 (NS-α) was recovered from the near-neutral baseline microcosm at pH 7.35, whereas bin112 (NS-α) was recovered from the acidified soil at pH 4.73. Three NS-δ MAGs, bin072, bin079, and bin088, were also recovered from the pH 4.73 soil. Thus, recovery from the acidified soil was not restricted to NS-α, and the genomic differences identified between NS-α and NS-δ should not be interpreted simply as a direct consequence of the pH at which individual MAGs were recovered. Rather, they may represent lineage-associated genomic characteristics that contribute to the contrasting ecological responses of these lineages during the transition from near-neutral to acidic soil conditions.

Such fine-scale differentiation is consistent with observations that pH preference cannot be inferred reliably from broad AOA phylogeny alone. Active AOA detected in acidic soils can be closely related to the nominally neutrophilic *Nitrososphaera viennensis* EN76 lineage [12]. Adaptation to acidic conditions may therefore emerge over relatively short evolutionary distances rather than requiring deep phylogenetic divergence [10,20]. Differences in gene content, sequence composition, genome organization, or gene regulation could generate ecologically distinct populations within closely related NS lineages.

An advantage of the present comparison is that the reconstructed NS genomes originated from the same soil system before and after ammonium-driven acidification rather than from unrelated field sites. In large-scale surveys, pH often covaries with nitrogen availability, organic matter, metal concentrations, vegetation, and other physicochemical properties, complicating the interpretation of pH-associated genomic or phylogenetic patterns [32,33,34]. The present system therefore provides a complementary framework for comparing NS lineages within a common soil background. Nevertheless, ammonium-driven acidification simultaneously alters nitrogen chemistry and ionic conditions [3,35]. The ecological differentiation between NS-α and NS-δ should therefore be interpreted in the context of the integrated environmental consequences of acidification rather than proton concentration alone [36,37].

More broadly, these findings emphasize that treating AOA as a single functional group may obscure substantial lineage-specific ecological variation. Closely related AOA lineages may respond differently to environmental change even when they share the same broad metabolic function of ammonia oxidation. Resolving such differentiation at finer phylogenetic and genomic scales is therefore important for understanding the stability and regulation of soil nitrification.

### 4.2. Comparative Functional Genomic Features of NS-α and NS-δ During Soil Acidification

Acidification imposes multiple physiological constraints on ammonia oxidizers. Decreasing pH lowers free NH_3_ availability, increases the energetic cost of intracellular pH and ion homeostasis, and may enhance oxidative and macromolecular stress [13,21,38,39]. Adaptation to acidic environments is therefore unlikely to depend on a single resistance determinant and is more plausibly achieved through coordinated mechanisms involving substrate acquisition, energy metabolism, stress defense, membrane properties, and cellular damage repair [16].

Functions involved in ammonium acquisition, P-II-mediated nitrogen regulation, and urease-mediated nitrogen utilization were detected in both NS lineages. These pathways may support metabolic flexibility as substrate conditions change, particularly when decreasing pH reduces free NH_3_ availability [13,40,41]. However, their occurrence in multiple NS-δ MAGs indicates that they cannot by themselves explain the contrasting ecological behavior of NS-α.

Respiratory energy metabolism and autotrophic carbon fixation showed a similar pattern. Genes associated with respiratory complexes, V/A-type ATPase, and the 3-HP/4-HB pathway were broadly distributed across both lineages. Previous studies have linked the acquisition of V-type ATPase operons to expansion of some AOA into acidic habitats [20]. Phylogenetic analysis of ATPase subunit D in the present study placed the retained bin079 homolog within the V-type-like group, whereas retained NS-α homologs grouped with A-type-like sequences. Thus, the present data do not support a preferential association of V-type ATPase subunit D with NS-α persistence under acidification. Moreover, the ATPase-related subunits retained in refined bin079 occur on separate contigs, so a complete continuous ATPase operon could not be reconstructed (Supplementary Table S7). The evolutionary and functional significance of ATPase variation therefore remains unresolved.

Oxidative-stress defense may represent another component of cellular stress management [22,42]. Catalase- and peroxiredoxin-associated genes were detected in both reconstructed NS-α MAGs, whereas a high-confidence SOD annotation was detected only in bin112; comparable antioxidant functions were also present in several NS-δ MAGs (Supplementary Tables S4 and S5). These observations do not support an NS-α-specific antioxidant repertoire. The genomic context of the catalase candidates was additionally examined and is summarized in Supplementary Table S7.

A notable DNA-repair-related pattern involved Mre11, HerA, NurA, and RadA. Mre11, HerA, and NurA were adjacent in both reconstructed NS-α MAGs, with the same transcriptional order (NurA–HerA–Mre11), whereas RadA occurred on separate contigs (Figure S1; Supplementary Table S8). HerA and NurA are established archaeal DNA-end-processing components and should not be interpreted as NS-α-specific innovations [43,44,45]. Consistent with this broader evolutionary context, Mre11, HerA, NurA, and RadA homologs were also identified in representative NS-α reference genomes, including the neutrophilic *Nitrososphaera viennensis* EN76 (Supplementary Table S6). Thus, the observed repertoire is more consistent with a broader NS-α-associated genomic feature than with an acid-specific adaptation. These gene detections describe genomic potential and do not demonstrate enhanced DNA-repair activity or a functional advantage under acidification.

Importantly, Rad50 was not detected in either reconstructed NS-α MAG and was detected only in NS-δ bin002 under the revised high-confidence MAG annotation criteria (Supplementary Tables S4 and S5). Although Rad50 homologs were recovered in whole-metagenome protein catalogs, these community-level hits could not be assigned to NS-α (Supplementary Table S3). Therefore, the Mre11–HerA–NurA–RadA repertoire observed in the NS-α MAGs should not be described as a complete classical Mre11–Rad50 double-strand-break repair pathway. Metagenomic detection also does not establish transcription, protein production, biochemical activity, or enhanced repair capacity [46,47]. Its potential relevance during soil acidification remains a hypothesis requiring functional validation.

Membrane and ion-homeostasis functions provide additional context. AIPS was retained in bin112 but was not detected as a high-confidence annotation in refined bin043, and it also occurred in several NS-δ MAGs (Supplementary Tables S4 and S5). Other membrane-lipid-associated functions likewise showed variable distributions. Genomic inventories alone are insufficient to infer actual membrane composition or proton permeability, and lipidomic analyses would be required to test membrane-remodeling hypotheses.

No high-confidence Na^+^/H^+^ antiporter or Trk/Ktr-type K^+^ transporter annotation was detected in either reconstructed NS-α MAG. Na^+^/H^+^ antiporters and K^+^ transport systems have been implicated in cytoplasmic pH and ion homeostasis in microorganisms, and related cation-transport systems have been proposed to contribute to pH regulation in acidophilic AOA [13,48,49]. In NS-δ, Na^+^/H^+^ antiporter annotations were detected in bin002, bin003, and bin079, whereas Trk/Ktr annotations were detected in bin008, bin024, and bin088 (Supplementary Tables S4, S5 and S7). These distributions do not indicate that either transporter class explains the contrasting ecological responses of NS-α and NS-δ. Whole-metagenome screening identified homologs of both transporter classes in the two source metagenomes, but those community-level hits could not be assigned to NS-α (Supplementary Table S3). Accordingly, the present data support only MAG-level non-detection in bin043 and bin112 and do not establish lineage-level absence or an alternative ion-homeostasis mechanism.

Taken together, the comparative genomic analysis identified both shared and differential features involving nitrogen acquisition, oxidative-stress-related functions, membrane and energy metabolism, and DNA-repair-related genes. Most of these functions were broadly distributed across NS-α and NS-δ, whereas the Mre11–HerA–NurA–RadA gene repertoire showed a notable distribution in the two reconstructed NS-α MAGs and was also identified in representative NS-α reference genomes. This broader distribution, including its occurrence in the neutrophilic *Nitrososphaera viennensis* EN76, is more consistent with an NS-α-associated genomic feature than with an acid-specific adaptation. These observations describe genomic potential and should not be interpreted as evidence of enhanced DNA-repair capacity, greater acid tolerance, or a direct mechanistic explanation for NS-α persistence. The MAG-based evidence nevertheless requires caution because genome quality varied among the reconstructed genomes. Although the Mre11–HerA–NurA–RadA pattern persisted after contig-level refinement, bin043 retained an estimated contamination of 15.4% and was excluded from the stringent contamination-filtered analysis, leaving only bin112 as an NS-α MAG representative. Thus, the consistency of this pattern cannot be established from the reconstructed MAGs alone. The additional NS-α reference genomes provide broader comparative support for its lineage association, but whether these DNA-repair-related genes contribute to physiological performance during soil acidification remains to be determined through controlled functional experiments.

Several limitations should be considered. First, because metagenomic sequencing was performed on one individual microcosm per condition, the present genome-resolved comparison does not provide a replicated statistical test of pH-associated lineage responses. Therefore, differences associated with the two source samples cannot be statistically separated from lineage-level genomic differences. Second, although contig-level refinement reduced estimated contamination, five MAGs still retained contamination > 10%, and genome completeness remained variable. A stringent sensitivity analysis excluding these MAGs retained only one NS-α genome (bin112), preventing an independent lineage-level assessment of candidate features shared by bin043 and bin112. Apparent gene absence may therefore reflect incomplete genome recovery, particularly in less complete MAGs, whereas gene presence in MAGs with residual contamination requires cautious interpretation. In addition, metagenomic annotation indicates genomic potential rather than in situ expression or biochemical activity. The observed genomic patterns should therefore be regarded as candidate lineage-associated features rather than definitive lineage-specific adaptations. Validation will require additional independently replicated metagenomes, broader sets of high-quality reference genomes, and complementary functional approaches. Future studies combining controlled pH experiments with SIP-based activity assessment and multi-omics analyses, together with targeted examination of ATPase operons, DNA-repair genes, and membrane lipids, will be required to determine whether these candidate traits directly contribute to NS-α/NS-δ ecological differentiation.

## 5. Conclusions

This study revealed genomic differentiation between NS-α and NS-δ ammonia-oxidizing archaea reconstructed from a near-neutral baseline microcosm (pH 7.35) and an acidified microcosm (pH 4.73), whereas most metabolic and stress-related functions were broadly distributed across both lineages. Following MAG refinement and reanalysis using high-confidence annotations, Mre11, HerA, NurA, and RadA were detected in both reconstructed NS-α MAGs, and homologs of these genes were also identified in representative NS-α reference genomes, including the neutrophilic *Nitrososphaera viennensis* EN76. This distribution is more consistent with a broader NS-α-associated DNA-repair-related genomic pattern than with an acid-specific adaptation. Na^+^/H^+^ antiporters and Trk/Ktr-type K^+^ transport systems were not detected within either reconstructed NS-α MAG, although MAG-level non-detection was not interpreted as definitive lineage-level absence. Given the use of one metagenomic microcosm per condition and the variable quality of the reconstructed MAGs, these results provide genomic context rather than a replicated test of acid-adaptation mechanisms. The physiological relevance of the DNA-repair-related pattern remains to be functionally tested.

## Figures and Tables

**Table 1:** Genomic characteristics of the reconstructed *Nitrososphaera*-affiliated metagenome-assembled genomes (MAGs).

MAG	Lineage	Soil pH	Completeness (%)	Contamination (%)	Contig N50 (bp)	Genome Size (Mb)	GC Content	Total Contigs	Max Contig Length (bp)	Min Contig Length (bp)
bin043	NS-α	7.35	74.0	15.4	1837	1.37	0.45	754	11,078	1001
bin112	NS-α	4.73	68.9	7.42	2168	1.52	0.56	755	12,449	1002
bin002	NS-δ	7.35	75.2	9.45	3295	1.83	0.35	671	24,543	1001
bin003	NS-δ	7.35	52.8	5.48	2330	1.44	0.39	651	19,848	1002
bin008	NS-δ	7.35	31.2	5.2	1540	1.22	0.27	780	7107	1001
bin013	NS-δ	7.35	29.5	2.8	1640	0.84	0.31	502	7040	1000
bin017	NS-δ	7.35	59.6	10.6	1680	1.40	0.34	782	15,619	1000
bin022	NS-δ	7.35	79.5	24.8	1559	1.84	0.42	1160	5391	1000
bin024	NS-δ	7.35	69.2	22.2	1522	4.77	0.38	3031	18,742	1000
bin072	NS-δ	4.73	43.7	6.8	1633	1.91	0.43	1114	40,880	1000
bin079	NS-δ	4.73	58.3	16.8	1782	2.72	0.37	1518	19,278	1000
bin088	NS-δ	4.73	51.9	7.96	1526	2.37	0.29	1526	7027	1000

Note: GC values in the current table are reported as genome-wide fractions. bin043 and bin112 were assigned to NS-α, whereas the remaining MAGs were assigned to NS-δ.

## Data Availability

The raw soil metagenomic sequencing data used in this study have been deposited in the NCBI Sequence Read Archive (SRA) under accession numbers SRR40513866 and SRR40513867. The refined MAG assemblies, predicted CDS/protein/GFF files, functional annotation outputs, ANI/AAI matrices, and software commands/parameters used for the revised analyses are provided in the Supplementary Data archive accompanying this article.

## Supplementary Materials

The following supporting information can be downloaded at: https://www.mdpi.com/article/10.3390/microorganisms14102250/s1. Figure S1: Genomic organization of the Mre11–HerA–NurA regions in the two refined NS-α MAGs. In bin043, NurA, HerA, and Mre11 are encoded consecutively on the positive strand of contig k121_1807088. In bin112, Mre11, HerA, and NurA are encoded consecutively on the negative strand of contig k121_1213075. Despite their opposite strand orientations, both regions show the same transcriptional gene order (NurA–HerA–Mre11), indicating a conserved and compact genomic organization; Table S1: Reference genomes and nucleotide sequences used in phylogenomic, amoA, and ATPase subunit D phylogenetic analyses; Table S2: Reassessment of amoA candidates after MAG refinement and revised functional annotation; Table S3: Metagenome-wide high-confidence screening of selected KO annotations in the near-neutral baseline and acidified metagenomes; Table S4: Distribution of 29 selected functional categories across the refined NS-α and NS-δ MAGs; Table S5: Gene- and subunit-level high-confidence KOfamScan annotations underlying the 29-category comparison; Table S6: DNA-repair-related gene distribution in representative NS-α reference genomes and reconstructed NS-α MAGs; Table S7: Genomic context validation of selected functional loci in refined NS-α and NS-δ MAGs; Table S8: Genomic organization and annotation support for Mre11, HerA, NurA, and RadA in the two reconstructed NS-α MAGs; Supplementary Data S1: Supplementary Commands and Software Parameters.

## Abbreviations

The following abbreviations are used in this manuscript:

AOA	Ammonia-oxidizing archaea
AOB	Ammonia-oxidizing bacteria
comammox	complete ammonia oxidizer
NS	*Nitrososphaera*
NT	*Nitrosotalea*
NP	*Nitrosopumilus*
NS-α	Nitrososphaera-related alpha lineage
NS-δ	Nitrososphaera-related delta lineage
AAI	Average amino acid identity
ANI	Average nucleotide identity
iTOL	Interactive Tree of Life
MAG	Metagenome-assembled genome
KO	KEGG Orthology
KEGG	Kyoto Encyclopedia of Genes and Genomes
3-HP/4-HB	3-hydroxypropionate/4-hydroxybutyrate
*amoA*	ammonia monooxygenase subunit A gene
AIPS	Archaetidylinositol phosphate synthase
ATPase	ATP synthase
ROS	Reactive oxygen species
SIP	Stable isotope probing
SOD	Superoxide dismutase
