# Supplementary material for: Comparative Genomics of Ecologically Differentiated Nitrososphaera-Related Ammonia-Oxidizing Archaea
Source: Microorganisms. 2026 Oct 4;14(10):2250. doi: 10.3390/microorganisms14102250 (PMC13707971; doi:10.3390/microorganisms14102250)
Supplement: Supplementary file 1 [file microorganisms-14-02250-s001.zip › Supplementary Figure S1.pdf]

Supplemental Figure

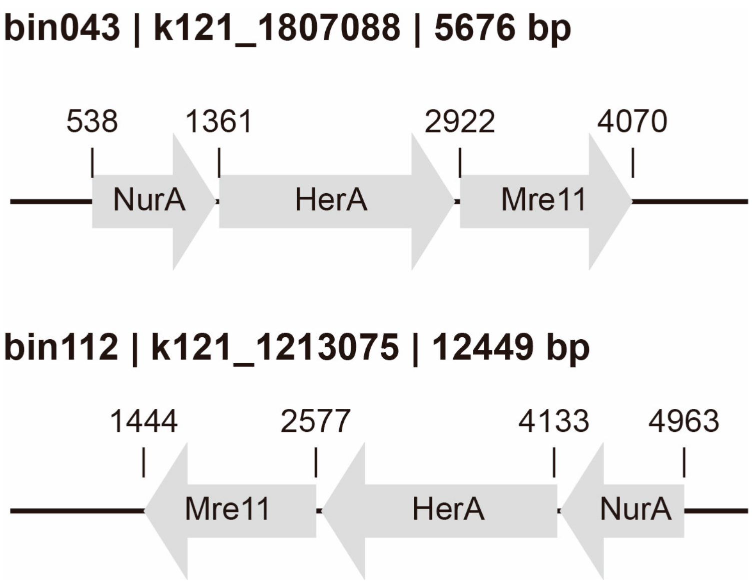

Figure S1. Genomic organization of the Mre11–HerA–NurA regions in the two refined NS-α MAGs. In bin043, NurA, HerA, and Mre11 are encoded consecutively on the positive strand of contig k121_1807088. In bin112, Mre11, HerA, and NurA are encoded consecutively on the negative strand of contig k121_1213075. Despite their opposite strand orientations, both regions show the same transcriptional gene order (NurA–HerA–Mre11), indicating a conserved and compact genomic organization.
